# Small molecular organic nanocrystals resemble carbon nanodots in terms of their properties†
†Electronic supplementary information (ESI) available. See DOI: 10.1039/c7sc02528a


**DOI:** 10.1039/c7sc02528a

**Published:** 2017-10-16

**Authors:** Syamantak Khan, Akshita Sharma, Sourav Ghoshal, Sanjhal Jain, Montu K. Hazra, Chayan K. Nandi

**Affiliations:** a School of Basic Science , Indian Institute of Technology Mandi , Mandi-175001 , HP , India . Email: chayan@iitmandi.ac.in; b Chemical Sciences Division , Saha Institute of Nuclear Physics , Homi Bhabha National Institute , Kolkata-700064 , WB , India . Email: h.montu@saha.ac.in

## Abstract

The most commonly observed phenomena in carbon nanodots (CNDs) are the strong excitation wavelength dependent multicolor fluorescence emission and the particle size distribution between 3–5 nm observed using a transmission electron microscope (TEM). However, it is not evident yet whether the emission originates from the particles observed using a TEM. In this article, we show that hydrothermal treatment of citric acid produces methylenesuccinic acid, which gives rise to hydrogen-bonded nano-assemblies with CND-like properties. While single crystal X-ray crystallography confirms the structure of methylenesuccinic acid, fluorescence correlation spectroscopy (FCS) confirms the presence of a molecular fluorophore with an average hydrodynamic diameter of ∼0.9 nm. This size is much smaller than the size of the particles observed using a TEM. We conclude that the particles observed using a TEM are the drying mediated nanocrystals of methylenesuccinic acid.

## Abstract 2

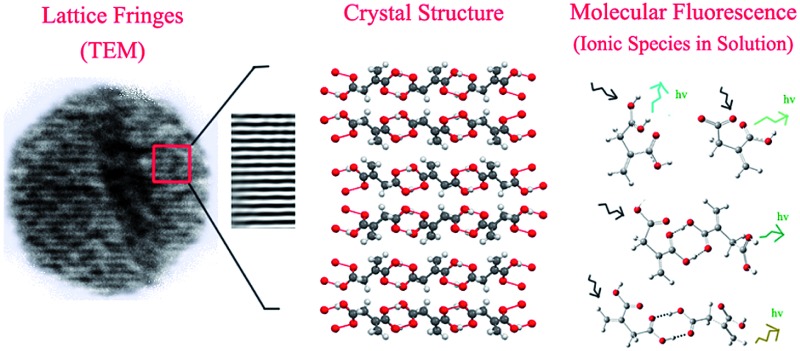
We show that hydrothermal treatment of citric acid produces methylenesuccinic acid, which gives rise to hydrogen-bonded nano-assemblies with CND-like properties.

Understanding the structure–function relationship of newly synthesized fluorescent materials is crucial for their applications in bioimaging, molecular sensors, optoelectronics and photovoltaics. High brightness with photo-stability, high aqueous solubility and low toxicity are the important criteria for their successful application. Carbon dots (CNDs), a new class of fluorescent nanomaterial in the carbon family, have been found to have such potential.^1^–^3^ Numerous experimental and theoretical studies^2^–^12^ considering quantum confinement,^2^ surface trap emission,^4^,^5^ variable oxidation states,^6^ aggregation induced emission^7^ and the presence of multichromophoric groups^8^ and crystalline and amorphous carbon clusters^9^,^10^ have been reported to explain the mechanism of the complex photoluminescence in CNDs. Nevertheless, the actual mechanism of the photoluminescence and chemical structure of the CNDs with a typical size of 3–5 nm remain elusive. Also, assuming a graphitic-like carbon core with a zero band gap, it is quite unlikely for the CNDs to have a high quantum yield (QY ≥ 90%) even after successful surface functionalization or chemical doping. Moreover, recent studies suggest that the small organic fluorophores that are formed at the first step of bottom-up CND synthesis determine the optical properties of the CND systems.^13^–^17^ From this perspective, the exact origin of the emission of any particle between ∼3–5 nm, which we observe using a TEM, becomes unclear and questionable. While both top-down (starting from graphene or carbon soot) and bottom-up (starting from small organic molecules) approaches have been proposed for the synthesis of CNDs with very similar optical properties, the very high QY of the CNDs produced by the bottom-up approach^16^ puts a question mark on their identity. Thus, a clear knowledge of their actual structure is crucial for the future progress of CND research.

Interestingly, citric acid (CA), which is a very well known molecule used for centuries to produce high QY fluorophores *via* a condensation reaction, has been used to synthesize CNDs in a bottom-up approach by pyrolysis. By solving the crystal structure using single crystal X-ray diffraction and measuring the diffusion coefficient using fluorescence correlation spectroscopy (FCS), we show that the CND-like properties originate from hydrogen-bonded nano-assemblies of methylenesuccinic acid (QY ∼ 1%) synthesized hydrothermally from citric acid (Fig. 1A). While single crystal X-ray crystallography confirms the structure of methylenesuccinic acid, FCS confirms the presence of a molecular fluorophore of size ∼0.9 nm in solution. From this observation we conclude that the particles observed using a TEM with an average diameter of ∼3–5 nm are the drying mediated hydrogen-bonded nanocrystals of methylenesuccinic acid. Therefore, the nanoparticles commonly observed under TEM do not confirm the formation of carbogenic CNDs with certainty. It should be noted here that 5-oxo-3,5-dihydro-2*H*-thiazolo[3,2-*a*]pyridine-3,7-dicarboxylic acid (TPDCA, QY ∼ 70%) is another bright fluorophore with an aromatic chromophore and is the major fluorescent component of a nitrogen and sulfur-doped CND.^15^ We found that similarly to methylenesuccinic acid, TPDCA also shows the phenomenon of nanocrystal formation.

**Fig. 1:**
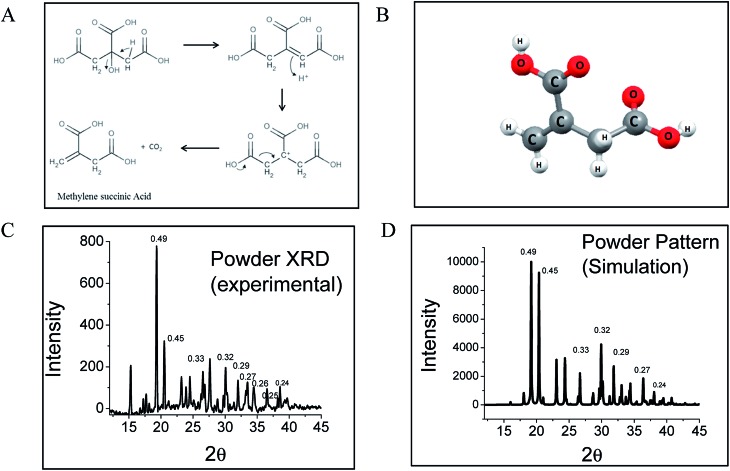
(A) Proposed reaction mechanism of the formation of methylenesuccinic acid from citric acid. At high temperatures and in slightly acidic media (acetic acid solvent), a dehydration step is followed by a decarboxylation step to produce methylenesuccinic acid. (B) The crystal structure shows an asymmetric unit with a molecular formula of C_5_H_6_O_4_, a derivative of citric acid. (C) The experimental powder XRD data show a nearly identical pattern to (D) the predicted simulation spectrum obtained from single crystal data.

Synthesis of methylenesuccinic acid and TPDCA was performed following the standard synthesis protocol of CNDs^15^ (see ESI†). As it is known that a higher temperature favors carbonization with a reduction of QY,^18^ we strategically employed a low-temperature synthetic procedure to avoid carbonization and to achieve a homogeneous product. High quality purified fluorophores were extracted from the product mixture by crystallization using a proper solvent. Thus, we confirm that the sample did not contain any carbonized particles. All the experiments presented in this manuscript were performed by dissolving the pure crystals back into water. Single crystal X-ray diffraction reveals an orthorhombic lattice and a small asymmetric unit with dimensions of 5.4565 Å × 11.5892 Å × 18.4914 Å and a volume of 1.169 nm^3^. The asymmetric unit contains a single methylenesuccinic acid molecule with a molecular formula of C_5_H_6_O_4_. It has one unsaturated double bond and two carboxylic acid units (Fig. 1B), which were obtained from the hydrothermal dehydration rearrangement of CA (Fig. 1A). The simulated spectrum obtained from the single crystal data (pure methylenesuccinic acid) has a pattern that nearly overlaps with that from the experimental powder XRD data (crude sample). Both of the spectra show some sharp peaks each corresponding to different *d*_*h*,*k*,*l*_ spacings (Fig. 1C and D). The similarity of the patterns proves that the crude sample is mostly homogeneous and consists mainly of methylenesuccinic acid. Nuclear Magnetic Resonance spectroscopy (NMR), Fourier transform infrared spectroscopy (FTIR) and High-Resolution Mass Spectroscopy (HRMS) also confirm the structure obtained from the single crystal data. The chemical shift values (*δ*) from the 1H NMR show two proton peaks at *δ* = 6.30 and *δ* = 5.82 corresponding to the protons attached to the sp^2^ carbon and a down-shifted peak at *δ* = 3.37 for the two protons attached to the sp^3^ carbon (Fig. S1†). The mass spectrum shows a single negative ion peak with an *m*/*z* = 129.058 corresponding to the deprotonated methylenesuccinic acid (Fig. S1†). The FTIR spectrum shows a broadened C–H vibration region indicating a strongly hydrogen-bonded structure and two C=O stretching bands for both the α and β-unsaturated carboxylic acids (Fig. S1†).

Therefore, from the above evidence, it could be predicted that the material is a small molecular entity at least in solution. To verify this further, we performed FCS, which is one of the best techniques for understanding the size of a fluorescence emitter. It is a single molecule fluorescence technique which directly probes the emitter diffusion with high accuracy.^19^ We performed the FCS measurement by calibrating the diffusion volume with a standard fluorophore (Atto488, diffusion coefficient (*D*) = 400 ± 50 μm^2^ s^–1^).^20^ The normalized autocorrelation curve, which nearly overlaps with the autocorrelation curve of Atto488, returns a value of the diffusion coefficient = 482 ± 50 μm^2^ s^–1^. This value corresponds to an approximate hydrodynamic diameter *D* = 0.9 nm, obtained using the Stokes–Einstein equation (Fig. 2A). The immediate question that arises is whether the observed nanoparticles (observed using TEM) are the real fluorescence emitters. Interestingly, when we used the same single fluorescent microcrystals for TEM, the nanoparticles were seen to have an average diameter of 3.6 nm and a lattice spacing of 0.21 nm, which is a common characteristic of CNDs (Fig. 2B). The selected area electron diffraction (SAED) pattern shows ring patterns indicating the polycrystalline nature of the sample (Fig. S2†). The diameters of the rings were measured to calculate the preferred crystallite orientations and the *d*_*h*,*k*,*l*_ spacings. Three major *d*_*h*,*k*,*l*_ spacings were found to be 0.12 nm, 0.20 nm and 0.33 nm. These are also found in the simulated single crystal X-ray and powder XRD data. A recent FCS study on CNDs has also found that the fluorescence emitter has a hydrodynamic diameter ∼1 nm.^21^ Nevertheless, to explain the observations from the TEM, the study used time-resolved electron paramagnetic resonance, which supported the existence of carbonized particles. In contrast, our observations from FCS and TEM originate from the same methylenesuccinic acid.

**Fig. 2:**
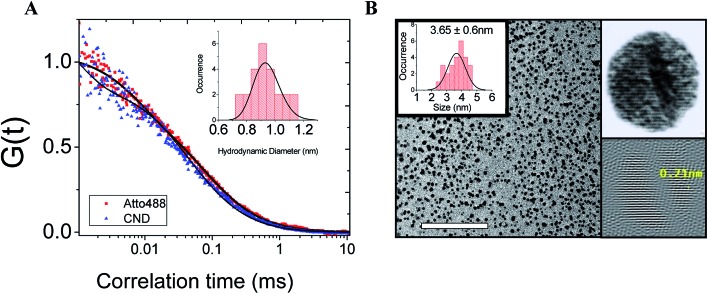
(A) Normalized autocorrelation curve which corresponds to an approximate hydrodynamic diameter of 8.9 ± 1.0 Å according to the Stokes–Einstein equation. (B) Transmission electron micrograph showing a uniform size distribution with a mean diameter of 3.65 nm. The scale bar is 100 nm. HRTEM shows the lattice fringes of the crystalline nanoparticles. Fast Fourier transform (FFT) analysis of the diffraction pattern shows a *d*-spacing of 0.21 nm between the lattice fringes.

Thus, it is evident that the nanoparticles observed using TEM have a much larger size than the fluorescent emitters seen using FCS. Thus these small emitters are either monomers or hydrogen-bonded dimers (or higher oligomers) of methylenesuccinic acid. The broad region of O–H stretching overlapping with the C–H stretching region of the FTIR spectrum (Fig. S1†) confirms the presence of doubly hydrogen-bonded dimers (DHBDs) of methylenesuccinic acid in solution. Therefore, we conclude that the particles observed using TEM are nanocrystallites of the small molecules which are formed during a drying mediated process as shown in Fig. 2B. The different lattice arrangements viewed through the (100), (010) and (001) planes give rise to different crystal planes and *d*-spacings (Fig. S3†). To verify whether similar chemical compounds could give rise to such TEM observations, we performed TEM experiments with commercially available methylenesuccinic acid and maleic acid. Interestingly, both of them show very similar crystalline nanostructures with identical *d*-spacings (0.21 nm) (Fig. S4†). We obtained a similar observation with TPDCA which is a bright fluorophore. TPDCA also formed uniform spherical nanocrystals with similar lattice fringes in a drying mediated process (Fig. S5†). It is noteworthy that CNDs have always been reported as spherical particles and the reason has not been understood to date. Here, the uniform spherical size distribution of the nanocrystals can be explained by surface energy minimization during the rapid drying process, as a sphere has the lowest surface area for a given volume. Thus energetically, spherical aggregates are most likely to form during the nano-crystallization process. Further studies are required for an in-depth understanding of the crystallization process resulting in a *d*-spacing of 0.21 nm. The actual optical properties of these nanocrystals are difficult to measure as they would require single particle optical measurements. Given that the actual optical properties of these nanocrystals are difficult to measure, we next performed quantum chemical calculations to understand the observed spectral changes.

It is worth mentioning here that both the obtained methylenesuccinic acid and commercially available acids show excitation wavelength dependent multicolor emission in solution as well as in the crystalline form (Fig. 3A and B and S6†). As the observed spectral changes were still not clear, we further performed quantum chemical calculations (Tables S1–S4 and Fig. S7–S9†). It is quite obvious that a carboxylic acid, depending on the p*K*_a_ value, undergoes protonation–deprotonation with a change in pH. We performed time-resolved area normalized emission spectroscopy (TRANES), which is one of the best methods to identify two conjugated emissive species. The TRANES data show a pH-dependent spectral migration through an isoemissive point at 390 nm (Fig. S10†) and the excitation spectra show an isosbestic point at ∼320 nm (Fig. 3A). These features confirm the presence of protonated/deprotonated species in the solution.^22^ As a result, we consider monomers, dimers and their conjugated ionic species for our calculations (see ESI†). The calculated binding energy for the double hydrogen-bonded dimers (DHBDs) in solution is ∼11 kcal mol^–1^, which supports the stability of the species in solution. The higher energy S_0_ → S_4_ electronic transitions (π → π*) for both the neutral monomer (∼207 nm) and two dimers (∼221–224 nm) are found to be more strongly allowed than the low-lying S_0_ → S_1_ transition (n → π*). Similarly, the S_0_ → S_2_ transition for the monomer (229 nm) and the S_0_ → S_3_ transitions for the other four DHBDs (∼222–234 nm) are mostly allowed π → π* transitions in nature (see ESI†). These results explain the observed increasing absorption intensity from 250 nm to 200 nm. Analysis of the molecular orbitals suggests that the conjugated π electrons in the C=C and C=O functional groups are giving rise to the higher energy transitions. On the other hand, the protonated/deprotonated ionic species, especially the dimers, result in the allowed charge transfer (CT) and pure π → π* and n → π* transitions from 329–240 nm. Interestingly, these transitions are not observed in the absorption spectra but are seen in the excitation spectra. The reduced oscillator strengths (by ∼one order of magnitude) of the ionic species in comparison to those of the neutral species and the relatively higher detection sensitivity of fluorescence compared to absorbance explain why we do not observe these transitions in the absorption spectra. Fig. 3C summarizes the most allowed transitions in a few selected species, as mentioned above.

**Fig. 3:**
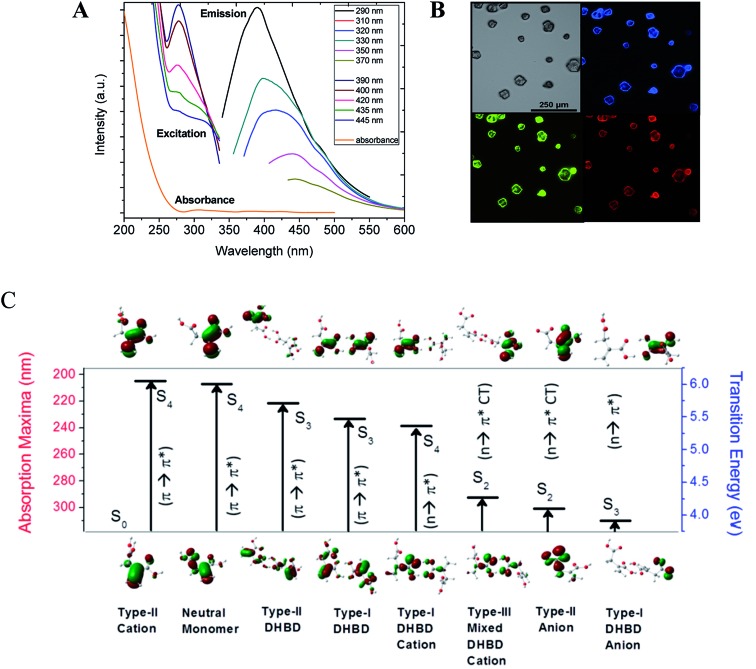
(A) The absorption, excitation and fluorescence spectra of methylenesuccinic acid. (B) Three-dimensional microcrystals grown at room temperature showed bright multicolor fluorescence under the microscope. (C) The most-probable transitions in different species predicted by quantum chemical calculations. The most allowed electron excitations occur to the higher energy states for methylenesuccinic acid, its dimers and the protonated/deprotonated ionic species. The corresponding molecular orbital for the most allowed transition has also been shown.

The emission spectra show the typical excitation dependent multicolor fluorescence with the emission maxima at 390 nm when excited at 290 nm (Fig. 3A). The very weak emission intensity (at ∼340–370 nm when excited at 200–250 nm) suggests that neither the neutral monomers nor the dimers are the main emissive species, rather the allowed emission (at 386 nm) occurs mainly from the monomeric anionic species that has an n → π* CT electronic excitation at 301 nm. This observation explains the commonly observed mismatch between the absorption and excitation spectra of CNDs. Overall, the higher excited state absorptions (S_3_ and S_4_) having higher transition probabilities are expected to be nearly non-emissive in solution, while absorption to the low-lying excited state (S_1_) is very low and the emission mainly occurs from here. A material like this will have a low extinction coefficient irrespective of its QY.

It is noteworthy that the emission maxima of these ionic species shifted significantly and distinct emission lines are visible in the photoluminescence spectra depending on the excited state protonation and deprotonation process. It is important to note here that a red shift in the emission maxima is evident (from 386 nm to 476 nm) in the dimers and their ions with a decrease in emission intensity. Therefore, we infer that excitation-dependent multicolor fluorescence can arise considering the co-existence of the neutral monomer, dimers and their ionic species. This knowledge is important as the concentration of CNDs often determines their optical properties, even enabling tuning of the emission wavelength.^23^,^24^ This has led to the theory of aggregation-induced emission of nano-clustered CNDs.^7^,^25^,^26^ Although our study provides theoretical evidence in support of aggregation-induced emission, it also experimentally confirms that the aggregates are formed only during the drying mediated process. Therefore, in contrast to some previous literature,^13^,^25^,^27^ we conclude that TEM analysis cannot confirm aggregation-induced emission in solution. Secondly, the fluorescence emitters are very small^21^ and free from aggregates, and finally, aggregation-induced emission is only relevant at a very high concentration or in the solid state.

The next immediate question which arises is whether it is reasonable to call the material ‘carbon nanodots’. It appears that in the literature the optical properties of CNDs are always more critically analyzed than their structures and new materials are classified as CNDs based on their common optical signatures. The assumption of a graphitic core has been widely accepted and even extended to heteroatom doping to increase the QY up to 99%.^28^–^30^ However, most of those are organic fluorophores and in some cases they even show excitation wavelength dependent fluorescence.^15^,^25^,^31^ Therefore, the concept of QY increase by heteroatom doping also appears to be severely misleading in the field of CNDs. Our results now show direct evidence for the formation of molecular aggregates in a spherical nanocrystallite shape that are often misunderstood as carbon nanoparticles when observed using TEM. This knowledge is very pivotal, as most of the previous studies have used TEM images to provide direct evidence for nanoparticle formation. The formation of a graphitic sp^2^ carbon core during high-temperature carbonization and in a top-down approach should not be ignored; nevertheless, thorough investigations with stronger evidence are extremely essential. Incorrectly attributing the optical properties of molecular fluorophores to these materials has created confusion in this field of research. The concepts of core/surface emission, size tuning or heteroatom doping need to be reviewed clearly in the context of molecular fluorophores.

In conclusion, we provide evidence that the bottom-up synthesis routes of CNDs may produce molecular fluorophores which can solely govern both their optical and chemical properties. We show that they can also form nanocrystallites mimicking carbogenic nanoparticles. Hence, the formation of carbogenic nanoparticles needs stronger experimental proof to avoid erroneous conclusions. On the one hand, this provides an excellent and facile route for synthesizing a bright fluorophore with unique optical properties, but on the other hand, researchers who are interested in actual carbon-based nanoparticles should be cautious in using bottom-up approaches for the same reason. At this point, it is also important to check if the photoluminescence of CNDs that are synthesized by top-down approaches has similar problems. Thus, the research field of CNDs needs to be addressed carefully to advance it in the right direction.

## Experimental and theoretical section

The materials and methods, Tables S1–S4 and Fig. S1–S10 can be found in the ESI.†

## Conflicts of interest

The authors declare no conflict of interest.

## Supplementary Material

Supplementary information
