# Supplementary material for: Small molecular organic nanocrystals resemble carbon nanodots in terms of their properties
Source: Chem Sci. 2017 Oct 16;9(1):175–80. doi: 10.1039/c7sc02528a (PMC5869300; doi:10.1039/c7sc02528a)
Supplement: Supplementary file 1 [file SC-009-C7SC02528A-s001.pdf]

Electronic Supplementary Material (ESI) for Chemical Science.
This journal is © The Royal Society of Chemistry 2017

Supporting Information

Small molecular organic nanocrystals resemble carbon nanodots in terms of their properties

Syamantak Khan[1], Akshita Sharma[1], Sourav Ghoshal[2], Sanjhal Jain[1], Montu K. Hazra[2], Chayan K. Nandi[1]**

[1]School of Basic Science, Indian Institute of Technology Mandi, Mandi-175001, HP, India
[2]Chemical Sciences Division, Saha Institute of Nuclear Physics, Homi Bhabha National Institute, Kolkata-700064, WB,

Contents:

Methods

Table S1-S4

Figure S1-S10

References

Methods

Synthesis:

Approx. 5g Citric acid ($C_6H_8O_7$) (CA) was dissolved in 7ml of acetic acid ($C_2H_4O_2$) to make a saturated solution. The mixture was heated at 180^0 C for 12 hours in a hydrothermal reactor. The product was filtered by Whatman filter paper and freeze-dried and stored at room temperature under vacuum or in a desiccator due to its moderate hygroscopic nature. To purify the product, 1ml solution was left unattended in 5ml beaker at 37^0C incubator for 7 days till crystals appear in mother liquor. The crystals were picked using a tip/needle and were washed in ethyl acetate to ensure no impurities stick to its surface. They were then dried to remove ethyl acetate and re-dissolved in water.

For synthesis of TPDCA, 0.025 mol powders mixtures of each citric acid anhydrous and L-cysteine were mixed in a beaker and heated up to 150 °C in an oven for 2 hours. The resulting orange colored solid obtained after 2 hours was dissolved in 25ml of cold deionized water and suctioned filtered carefully , filtrate was collected .The TPDCA crystals were obtained by heating the filtrate up to 90°C and there after keeping for crystallization at room temperature for 24 hours. The obtained TPDCA crystals were washed several times with cold water to remove any impurity before further analysis.

Transmission Electron Microscopy (TEM)

The particle size and dispersity of the synthesized samples were checked using a TECNAI G2 200 kV TEM (FEI, Electron Optics) electron microscope with 200 kV input voltage. TEM grids were prepared by placing 5 µL diluted and well sonicated sample solutions on a carbon coated copper grid and evaporated the solution at room temperature completely. Precautions were taken to avoid contamination from various sources.

Atomic Force Microscopy (AFM)

AFM analysis of the synthesized samples for particle size determination was carried out using a Digital Instruments Bruker AFM. Standard Veeco tapping mode silicon probes were used for scanning the samples. Typically, samples were dried on silicon substrate for overnight. Once dried, samples were placed on the AFM stage and scanned. Pertinent scanning parameters were as follows: Resonant frequency (probe): 60-80 kHz; Tip velocity for all measurements are: (4 µm/s for 2 µm), (15 µm/s for 5

μm), (30 μm/s for 10 μm). Aspect ratio: 1:1; Resolution: 512 samples/line, 256 lines. Precautions were taken to avoid artifacts.

Fourier Transform Infrared Spectroscopy (FTIR)

FTIR spectra of samples were measured using an Agilent FTIR spectrophotometer equipped with a horizontal attenuated total reflectance (ATR) accessory containing a zinc selenide crystal and operating at 4 cm^{-1} resolution. The use of the spectral subtraction provided reliable and reproducible results.

Nuclear Magnetic Resonance (NMR)

^{1}H and ^{13}C NMR spectra were recorded on Joel JNM ECX-500 FT-NMR spectrometer using $CDCl_3$ or D_2O as solvent and TMS as internal standard. Data are reported as follows: chemical shift in ppm (δ), multiplicity (s = singlet, d= doublet, br = broad singlet, m = multiplet), coupling constant J (Hz) integration, and interpretation. ESI-TOF HR-MS spectra were recorded by using Bruker Maxis impact instrument.

High Resolution Mass Spectroscopy (HRMS)

High Resolution Mass spectral data was recorded using Bruker Daltonik GmbH (Model – Impact HD, USA) instrument in positive mode by injecting 1mL of diluted samples in capillary at 3500 V. Active mode scanning of spectra has been recorded at 2000 V charging voltage from 250m/z to 1250m/z. (Nebulized pressure = 0.3Bar, dry gas = 4L/min at 200°C temperature dry heater).

X-ray Diffraction (XRD)

Powder X-ray experiment was carried out using powder X-ray diffractometer which is built on 9 KW rotating anode x-ray generator with NaI Scintillation counter detection system. Copper anode were used as the target material with fine focus filament as the cathode. The sample was freeze dried to prepare dry powder before XRD experiment.

Single crystal X-ray data were collected on Agilent Super Nova diffractometer, equipped with multilayer optics monochromatic dual source (Cu and Mo) and Eos CCD detector, using Mo-Kα (0.71073 Å) radiation at temperature 150 K. Data acquisition, data reduction and analytical face-index based absorption correction were performed using the program CrysAlisPRO.6 The structure was solved

by Direct methods with ShelXS7 program and refined on F2 by full matrix least squares techniques with ShelXL7 program in Olex2 (v.1.2) program package.

Steady state and Time Resolved Fluorescence Spectroscopy

Steady state fluorescence was measured using Horiba Fluorolog-3 Spectrofluorometer and Cary Eclipse Fluorescence Spectrophotometer by Agilent Technology. All the experiments were performed at room temperature. The fluorescence was measured in 1 ml quartz cuvette. The fluorescence lifetime and time resolved emission spectra (TRES) were measured using Horiba scientific Delta Flex TCSPC system with Pulsed LED Sources. Ludox has been used to obtain IRF for de-convolution of the spectral value. The photon decays in different channels were fitted bi/tri-exponentially with a chi-squared value <1.2 in order to calculate fluorescence lifetime. TRES was plotted by taking appropriate slice for each case, for instance, 50 slices of the 3D plot at 13 channel (~0.35ns) interval. Approximately 3ml solution has been used for all measurements. TRANES were calculated from TRES data by normalizing the area under the curves. It was done simply by taking integration of the emission spectra followed by normalization of the integration curves. Finally the TRANES were obtained by differentiation of those normalized integration curves.

Fluorescence Correlation Spectroscopy:

Approx. 50µl sample solutions (~nanomolar concentrations) were drop-casted on cleaned glass coverslips and placed on an inverted confocal microscope (A1R+, Nikon, Japan) using a 1.4 NA oil-immersion 60X objective. A 488 nm gas laser was used to excite the sample at room temperature (25 °C) using appropriate dichroic and filters in the optical path. The emission beam was directed through a side-port of the microscope delivering it to a Hybrid Photomultiplier Detector Assembly single photon counting module (PicoQuant GmBh. Berlin, Germany). The fluorescence fluctuations were analyzed within a point region of interest (ROI) of the sample. The confocal volume (~1 femtoliter) was calibrated with standard fluorophore (Atto488) with a known diffusion coefficient. All data were analysed (fitting an autocorrelation function for calculating diffusion coefficients) using the SymPhoTime 64 software (PicoQuant GmBh).

Computational Methodology:

To understand the detail photo-physics of the methyl-succinic acid in aqueous phase, we perform quantum chemical calculations, especially, to predict both the absorption and emission spectra in aqueous phase. Gaussian-09 suite of program and the time dependent density functional theory (TDDFT) in conjunction with 6-31G+(d,p) basis set has been used to carry out all the quantum chemistry calculations presented here.[1] To predict absorption spectra in the aqueous phase, the B3LYP functional has been selected.[2] Similarly, the long range corrected CAMB3LYP and WB97XD functionals have been selected to predict the $S_1 \rightarrow S_0$ emission spectra.[3] Selection of these functionals is based upon their reliabilities in predicting both the absorption and emission spectra of various species, as reported in the literature.[2-7] Both the linear response (LR) and state specific (SS) formalisms/methods have been used to account the solvent effect through Polarizable Continuum Model (PCM).[3] It is to be noted here that although the SS-PCM method is expected to more reliable than LR-PCM method in consideration with the balanced description of the equilibrium and nonequilibrium solvation energies, nevertheless, the main drawback of the SS-PCM method is that it is highly sensitive to the change of dipole moment during the External-Iteration process and a slight change of the dipole moments (≥ 0.5 Debye) in the External-Iteration process overestimates the emission spectra significantly.[6] In this scenario, the standard LR-PCM method predicted data, which are qualitatively quite accurate, have been taken into account.[6-7] It is seen from our calculations that both the CAMB3LYP and WB97XD functionals are consistent to each other in predicting the $S_1 \rightarrow S_0$ emission spectra. Nevertheless, to keep our discussion simple in the text, we only highlight results predicted at the WB97XD functional.

Table S1: The TD-DFT/PCM-WB97XD/6-31+G(d,p) level optimized geometries of the neutral methylene succinic acid monomer, doubly hydrogen-bonded dimers (DHBDs) and their ionic species as shown in the Figure S7- S9 in the first electronically excited state (S_1).

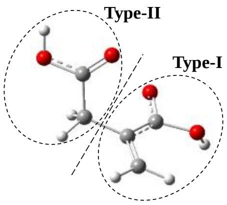 **Neutral Monomer**	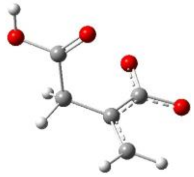 **Type-I Anion**	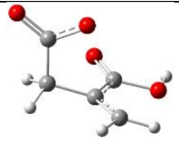 **Type-II Anion**	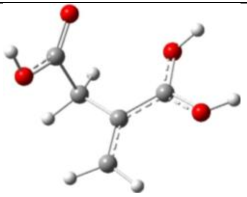 **Type-I Cation**
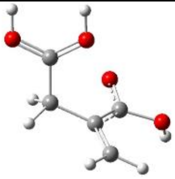 **Type-II Cation**	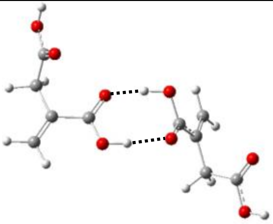 **Type-I DHBD**	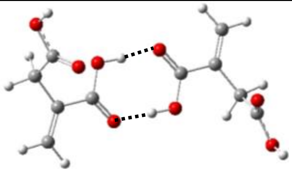 **Type-I DHBD Tautomer**	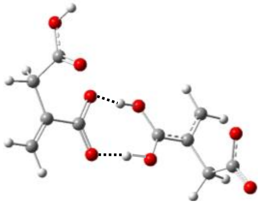 **Type-I DHBD Anion**
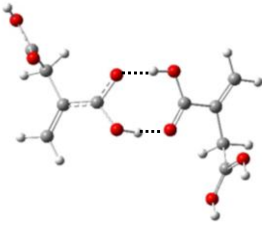 **Type-I DHBD Cation**	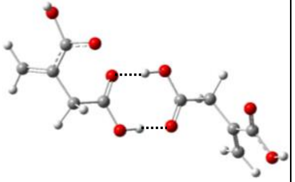 **Type-II DHBD**	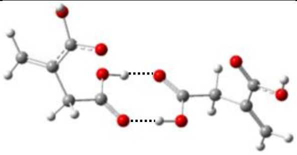 **Type-II DHBD Tautomer**	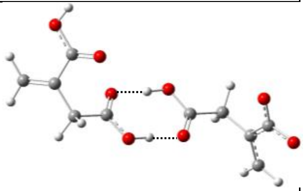 **Type-II DHBD Anion**
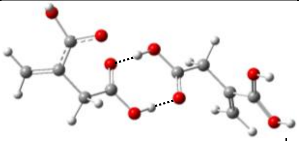 **Type-II DHBD Cation**	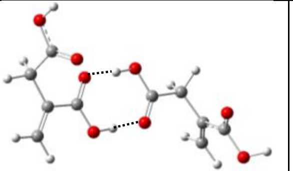 **Mixed DHBD**	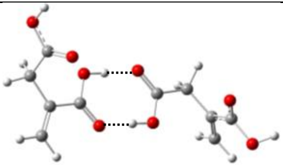 **Mixed DHBD Tautomer**	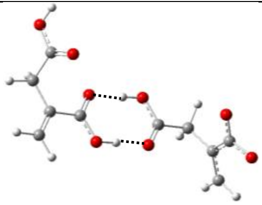 **Type-III Mixed DHBD Anion**
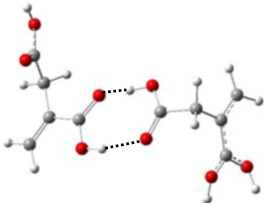 **Type-III Mixed DHBD Cation**	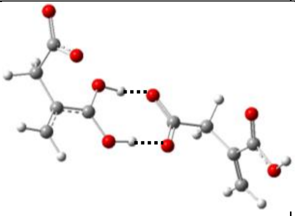 **Type-IV Mixed DHBD Anion**	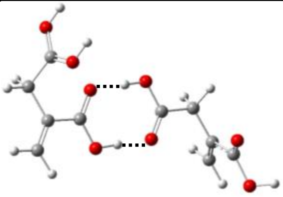 **Type-IV Mixed DHBD Cation**	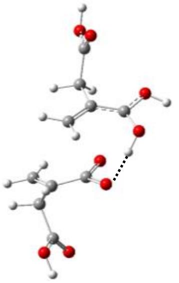 **Ionic Pair**[a]

[a]Single hydrogen transferred ionic pair has been optimized in the first electronically excited state. We did not find any such neutral ionic pair in the ground electronic state.

Table S2: Summary of transitions of different species in the PCM-B3LYP/6-31+G(d,p) level of calculations.

Species	$S_0 \rightarrow S_1$	$S_0 \rightarrow S_2$	$S_0 \rightarrow S_3$	$S_0 \rightarrow S_4$
**Neutral Monomer**	254.56 (0.0001)[a]	229.27 (0.0200)[a]	209.74 (0.0002)[a]	207.07 (0.3153)[a]
**Type-I Anion**	288.41 (0.0001)[a]	266.98 (0.0008)[a]	236.16 (0.0252)[a]	232.16 (0.0017)[a]
**Type-II Anion**	316.25 (0.0081)[a]	300.71 (0.0301)[a]	277.32 (0.0009)[a]	249.80 (0.0005)[a]
**Type-I Cation**	303.70 (0.0057)[a]	238.09 (0.0497)[a]	233.26 (0.2232)[a]	208.52 (0.0013)[a]
**Type-II Cation**	254.61 (0.0050)[a]	247.50 (0.0110)[a]	236.75 (0.0809)[a]	204.93 (0.2835)[a]
**Type-I DHBD**	243.86 (0.0000)[a]	241.28 (0.0000)[a]	233.53 (0.0528)[a]	233.14 (0.0027)[a]
**Type-I DHBD Tautomer**	244.86 (0.0022)[a]	242.07 (0.0001)[a]	232.39 (0.0694)[a]	231.95 (0.0003)[a]
**Type-I DHBD Anion**	328.71 (0.0071)[a]	316.16 (0.0041)[a]	309.86 (0.0179)[a]	299.53 (0.0078)[a]
**Type-I DHBD Cation**	254.64 (0.0064)[a]	249.46 (0.0008)[a]	241.17 (0.0041)[a]	238.59 (0.0154)[a]
**Type-II DHBD**	254.46 (0.0001)[a]	254.37 (0.0001)[a]	221.69 (0.0403)[a]	221.07 (0.0403)[a]
**Type-II DHBD Tautomer**	254.37 (0.0002)[a]	254.35 (0.0002)[a]	224.46 (0.0993)[a]	223.68 (0.0455)[a]
**Type-II DHBD Anion**	305.02 (0.0000)[a]	288.20 (0.0004)[a]	281.12 (0.0000)[a]	272.92 (0.0000)[a]
**Type-II DHBD Cation**	301.65 (0.0014)[a]	286.65 (0.0028)[a]	284.02 (0.0011)[a]	267.67 (0.0026)[a]
**Mixed DHBD**	254.49 (0.0001)[a]	243.33 (0.0001)[a]	232.85 (0.0209)[a]	220.99 (0.0091)[a]
**Mixed DHBD Tautomer**	254.33 (0.0002)[a]	244.05 (0.0010)[a]	231.45 (0.0259)[a]	226.83 (0.0056)[a]
**Type-III Mixed DHBD Anion**	313.71 (0.0002)[a]	288.65 (0.0010)[a]	288.17 (0.0004)[a]	279.90 (0.0001)[a]
**Type-III Mixed DHBD Cation**	302.73 (0.0001)[a]	292.38 (0.0075)[a]	282.34 (0.0000)[a]	269.33 (0.0039)[a]
**Type-IV Mixed DHBD Anion**	326.36 (0.0078)[a]	309.89 (0.0245)[a]	308.39 (0.0021)[a]	294.57 (0.0000)[a]
**Type-IV Mixed DHBD Cation**	254.40 (0.0001)[a]	254.06 (0.0067)[a]	248.01 (0.0075)[a]	339.32 (0.0029)[a]

[a]Calculated oscillator strengths in the PCM-B3LYP/6-31+G(d,p) level of calculations.

Table S3: TD-DFT emission energies [$S_1 \rightarrow S_0$ (ΔE_{em}, nm)] of the neutral methylene succinic acid monomer, doubly hydrogen-bonded dimers (DHBDs) and their ionic species as shown in the Figure S7-S9 at the PCM-CAM-B3LYP/6-31+G(d,p) and PCM-WB97XD/6-31+G(d,p) levels of calculations.

Species (First Excited State)	Emission Energies (ΔE_{em}, nm)			
	CAM-B3LYP/6-31+G(d,p)		WB97XD/6-31+G(d,p)	
	LR-PCM	SS-PCM	LR-PCM	SS-PCM
Neutral Monomer	331.72	357.45	336.63	365.54
Type-I Anion	421.69	448.46	430.76	464.71
Type-II Anion	377.58	*a*	385.56	*a*
Type-I Cation	340.84	*a*	333.22	*a*
Type-II Cation	329.01	354.69	338.76	365.13
Type-I DHBD	361.88	390.02	367.17	401.19
Type-I DHBD Tautomer	347.67	374.80	353.20	381.57
Type-I DHBD Anion	455.49	*a*	476.83	*a*
Type-I DHBD Cation	356.87	405.87	364.25	417.25
Type-II DHBD	328.58	354.32	336.39	364.42
Type-II DHBD Tautomer	330.50	356.40	335.67	361.24
Type-II DHBD Anion	418.49	441.51	430.23	461.46
Type-II DHBD Cation	328.88	357.74	336.80	364.83
Mixed DHBD	328.68	354.56	336.76	364.83
Mixed DHBD Tautomer	329.38	355.48	336.72	364.27
Type-III Mixed DHBD Anion	419.35	442.85	427.35	457.41
Type-III Mixed DHBD Cation	359.59	*a*	345.91	*a*
Type-IV Mixed DHBD Anion	455.07	*a*	473.62	*a*
Type-IV Mixed DHBD Cation	329.60	357.96	337.14	367.80
Ionic Pair	652.37	*a*	685.89	*a*

[a]The SS-PCM stands for State Specific Polarization Continuum Model and LR-PCM stands for Linear Response Polarization Continuum Model. The LR-PCM predicted data have been taken into account when the SS-PCM method overestimates the $S_1 \rightarrow S_0$ emission maxima, as mentioned above.

Table S4: The binding energies of double hydrogen-bonded dimers (DHBDs) formed from methylene succinic acid monomer at the PCM-B3LYP/6-31+G(d,p) level of calculations. For a particular DHBD, the binding energy has been calculated by subtracting the zero point energy corrected electronic energy of the DHBD from the total energy of neutral monomers formed the same DHBD.

**Double hydrogen-bonded dimer (DHBD)**	**Binding Energy (kcal/mol)**
Type-I DHBD	-10.77
Type-II DHBD	-10.28
Mixed DHBD	-10.58

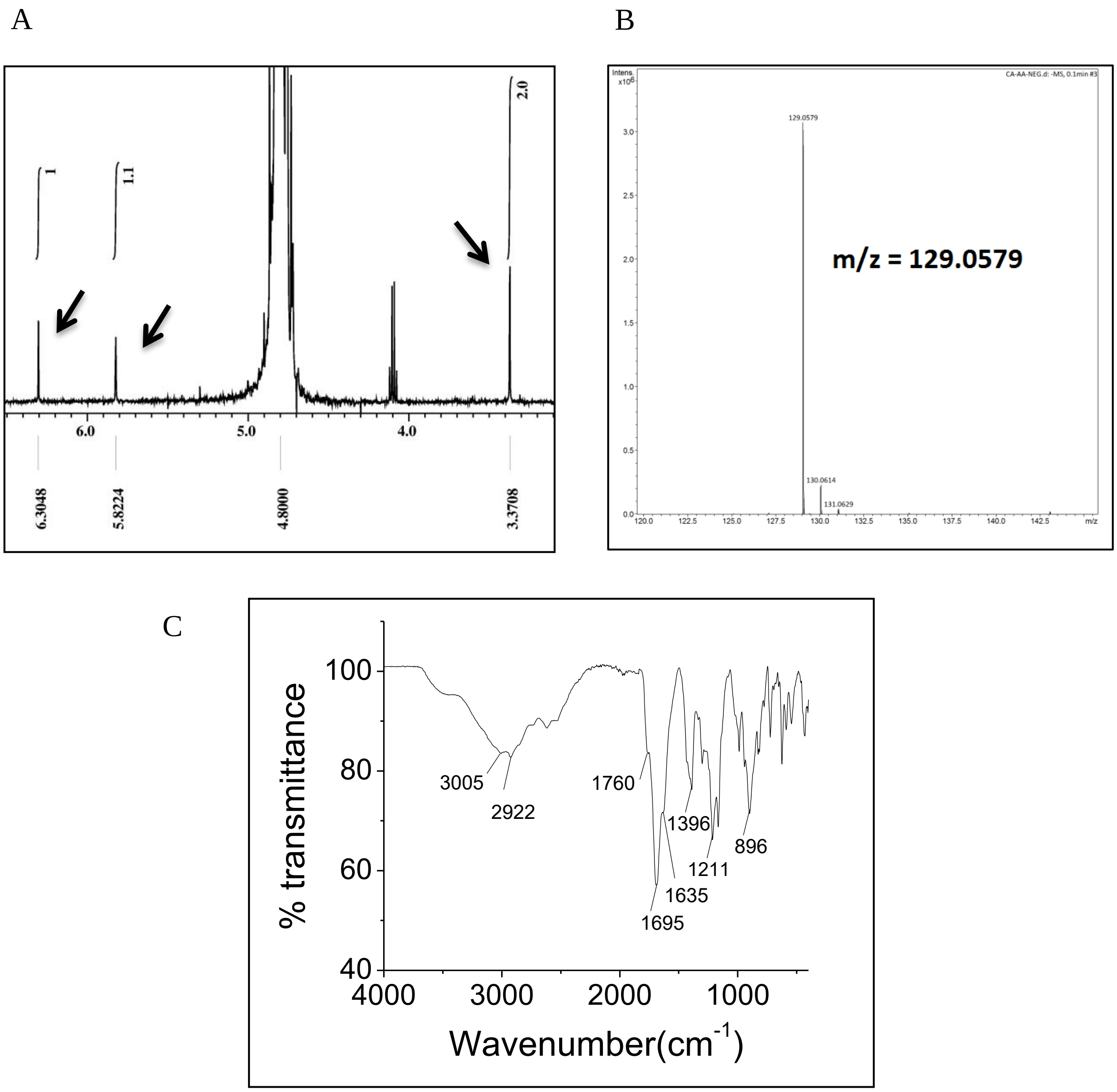

Figure S1. (A) ^{1}H NMR (B) HRMS and (C) FTIR shows signature of methylene succinic acid. ^{1}H NMR shows two proton peaks at $\delta = 6.30$ and $\delta = 5.82$, corresponding to the protons attached with sp^2 carbon and a down-shifted peak at $\delta = 3.37$ for two protons attached with sp^3 carbon. Mass spectra shows a single negative ion with m/z = 129.058 which is the deprotonated methylene succinic acid. FTIR shows broadened O-H stretching overlapped with C-H vibration, which arises from strong H-bonding in solid state.

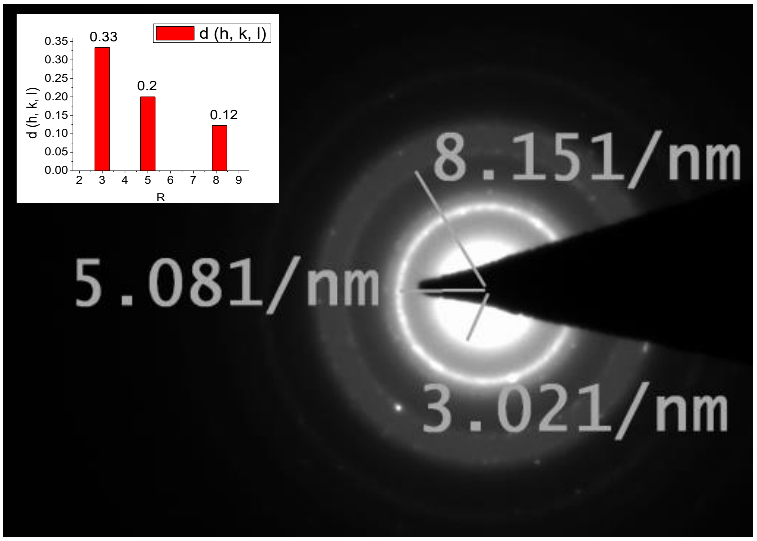

Figure S2. Electron diffraction pattern of selected area diffraction (SAED) show ring patterns indicating the polycrystalline nature of the sample. The diameters of the rings were measured to calculate the preferred crystallite orientations and $d_{h, k, l}$ spacing. Three major $d_{h,k,l}$ were found to be 0.12nm, 0.2 nm and 0.33nm

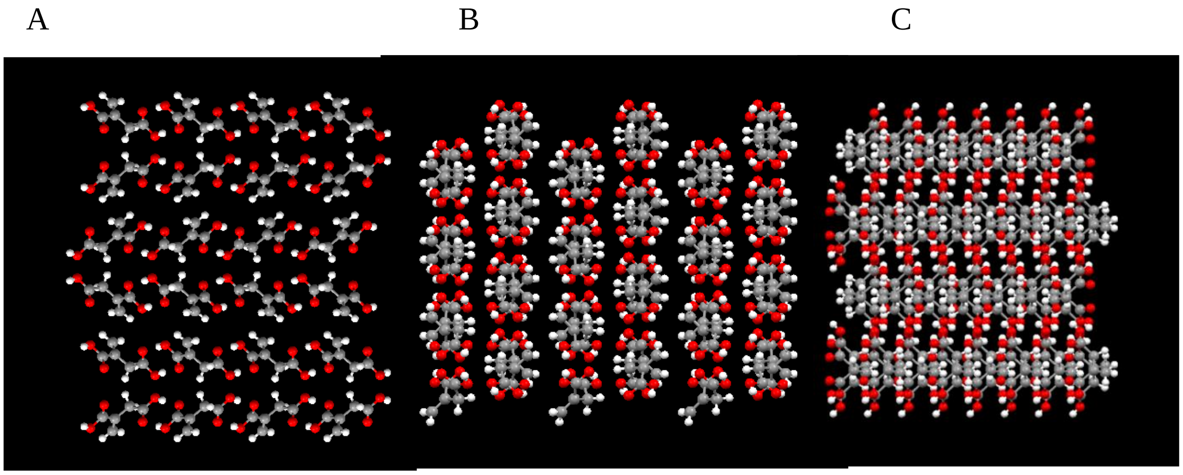

Figure S3. (A-C) The hydrogen bonded crystal lattice arrangement viewed along x, y, and z-axis respectively.

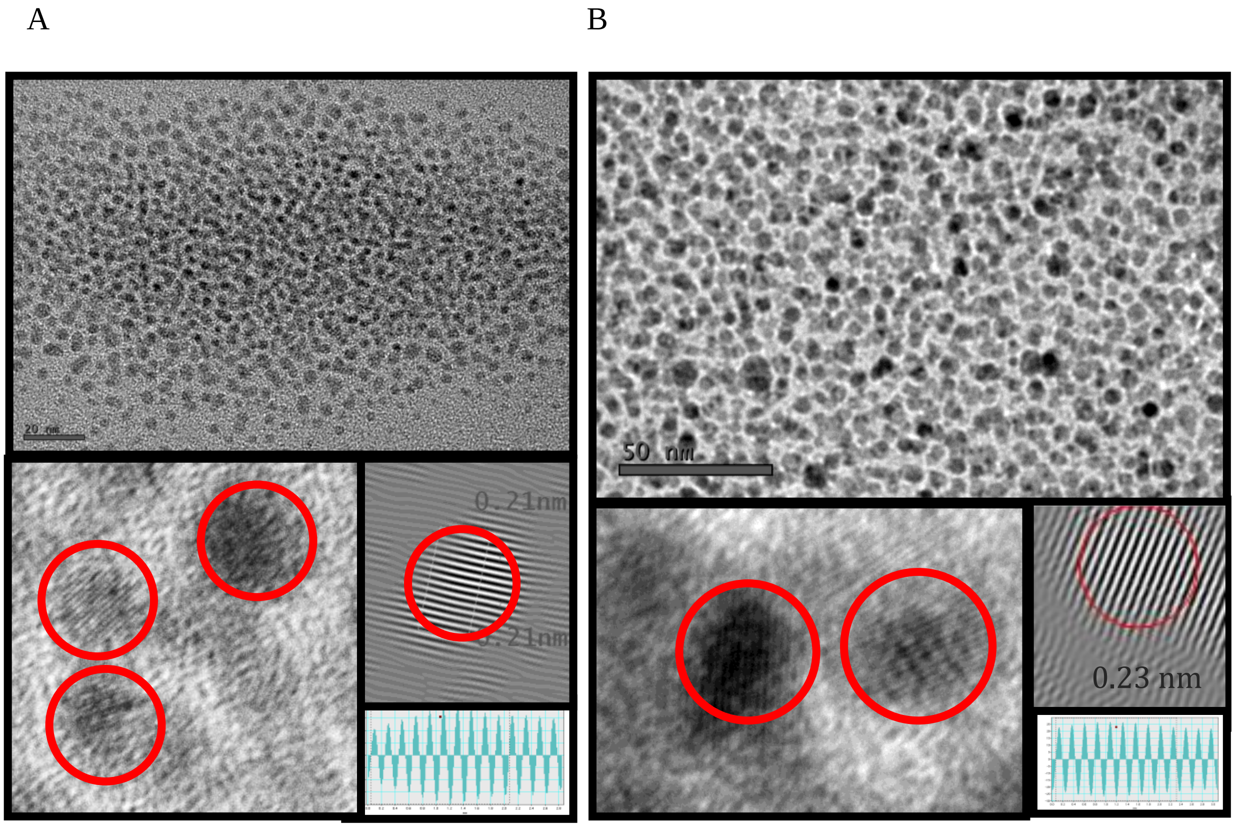

Figure S4 Transmission electron microscopic image of (A) methylene succinic acid and (B) maleic acid nanocrystals. A concentration of 10mg/ml was used for drop-casting on the carbon coated copper grid. Nanocrystals with spherical morphology, narrow particle size distribution (3-6nm) and a crystal lattice with 0.21nm / 0.23nm $d_{h,k,l}$ spacing (which is commonly attributed to graphitic carbon) were observed.

A

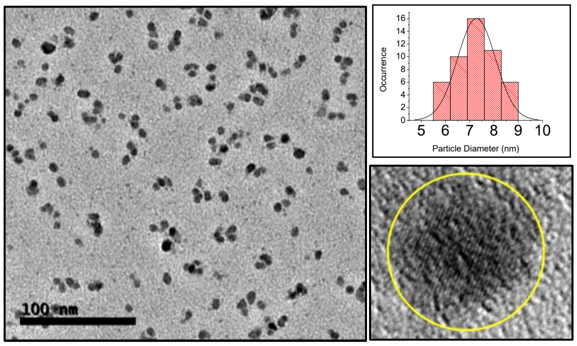

B

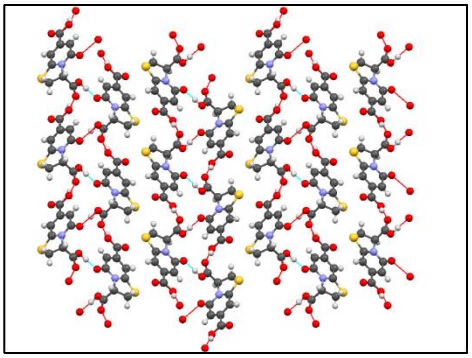

Figure S5: Nanocrystals of TPDCA (a) Nanocrystals formed on the TEM grid during drop drying process looks similar to commonly observed carbon nanoparticles. The nanocrystals have an average diameter of 7.5nm and shows crystallinity in HRTEM. (b) Hydrogen bonded assembly of TPDCA as observed in the crystal structure.

(A)

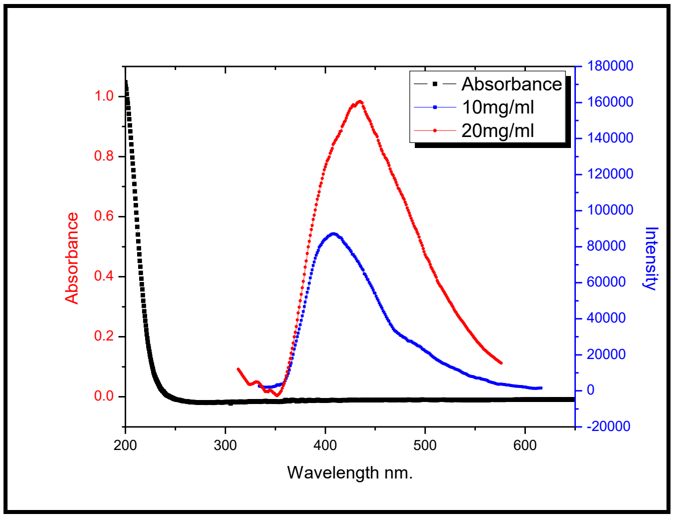

(B)

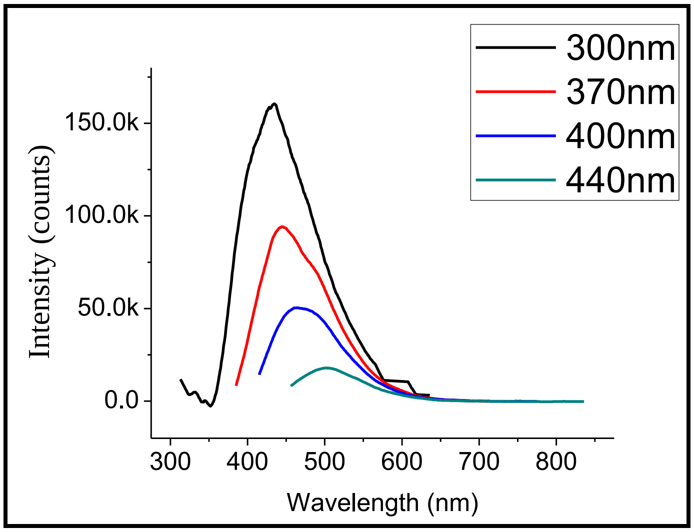

(C)

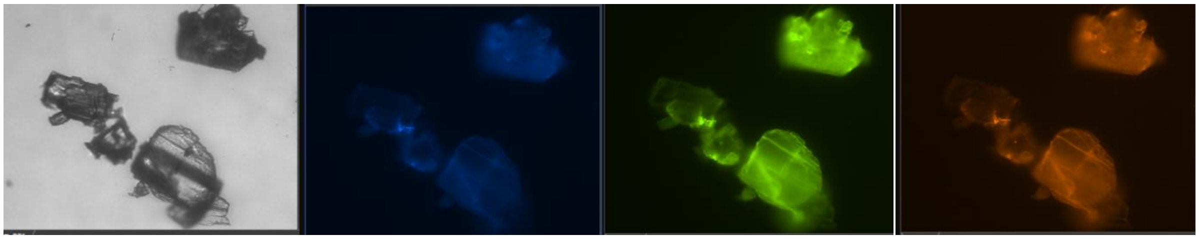

Figure S6 Optical properties of commercially available methylene succinic acid. (A) Absorbance and fluorescence spectra. A red shift was observed with increasing concentration indicating increased emission from hydrogen-bonded dimers. (B) Excitation dependent fluorescence as commonly observed in CNDs. (C) Solid state multicolour fluorescence as observed under fluorescence microscope.

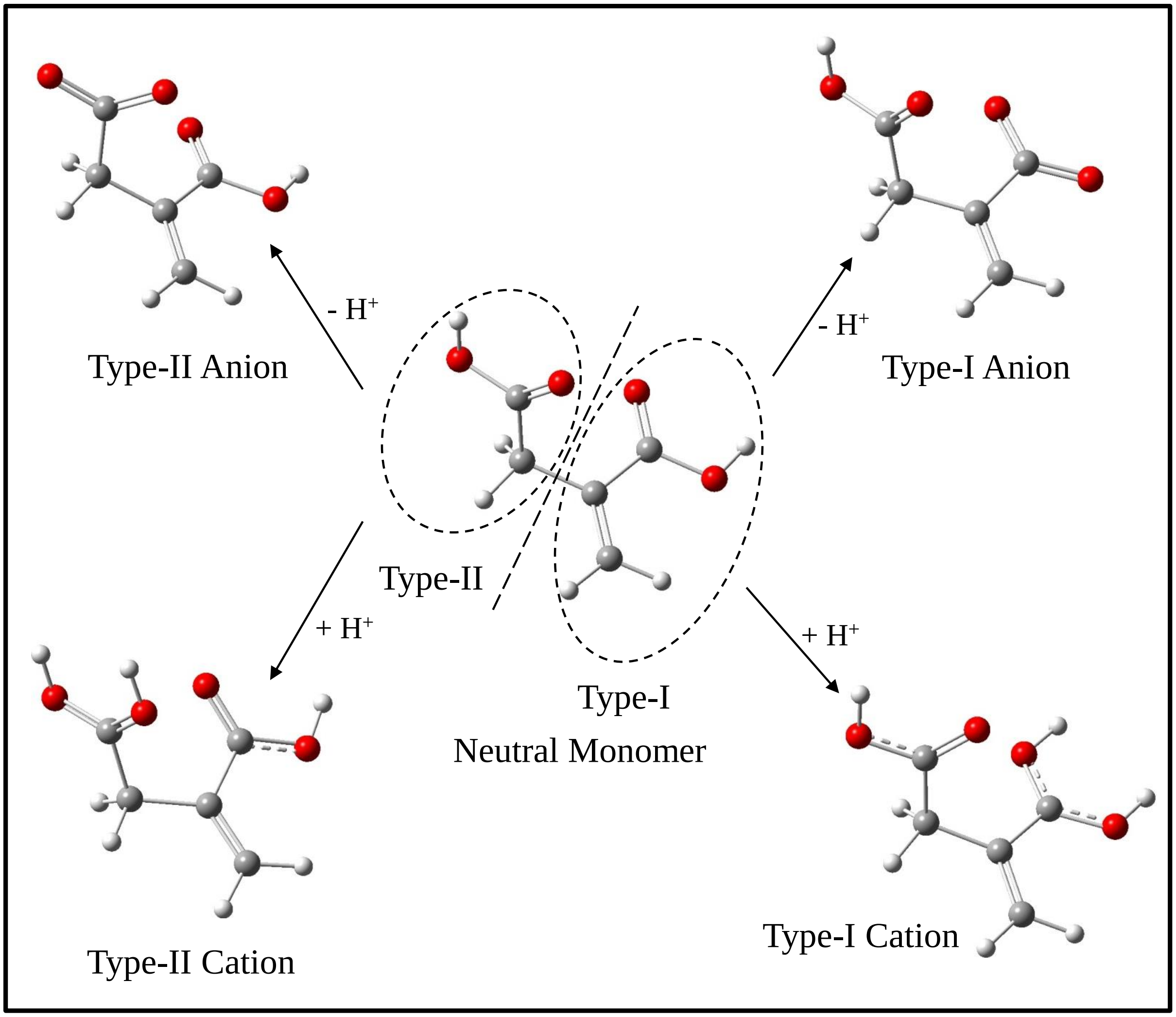

Figure S7: The PCM-B3LYP/6-31+G(d,p) level optimized geometries of neutral monomer and its ionic species. It is to be noted here and in the other figures and tables that the two COOH groups (Type-I and Type-II) of the methylene succinic acid monomer are not equivalent to each other, as the adjacent functional groups or local environments of each COOH functional group are different.

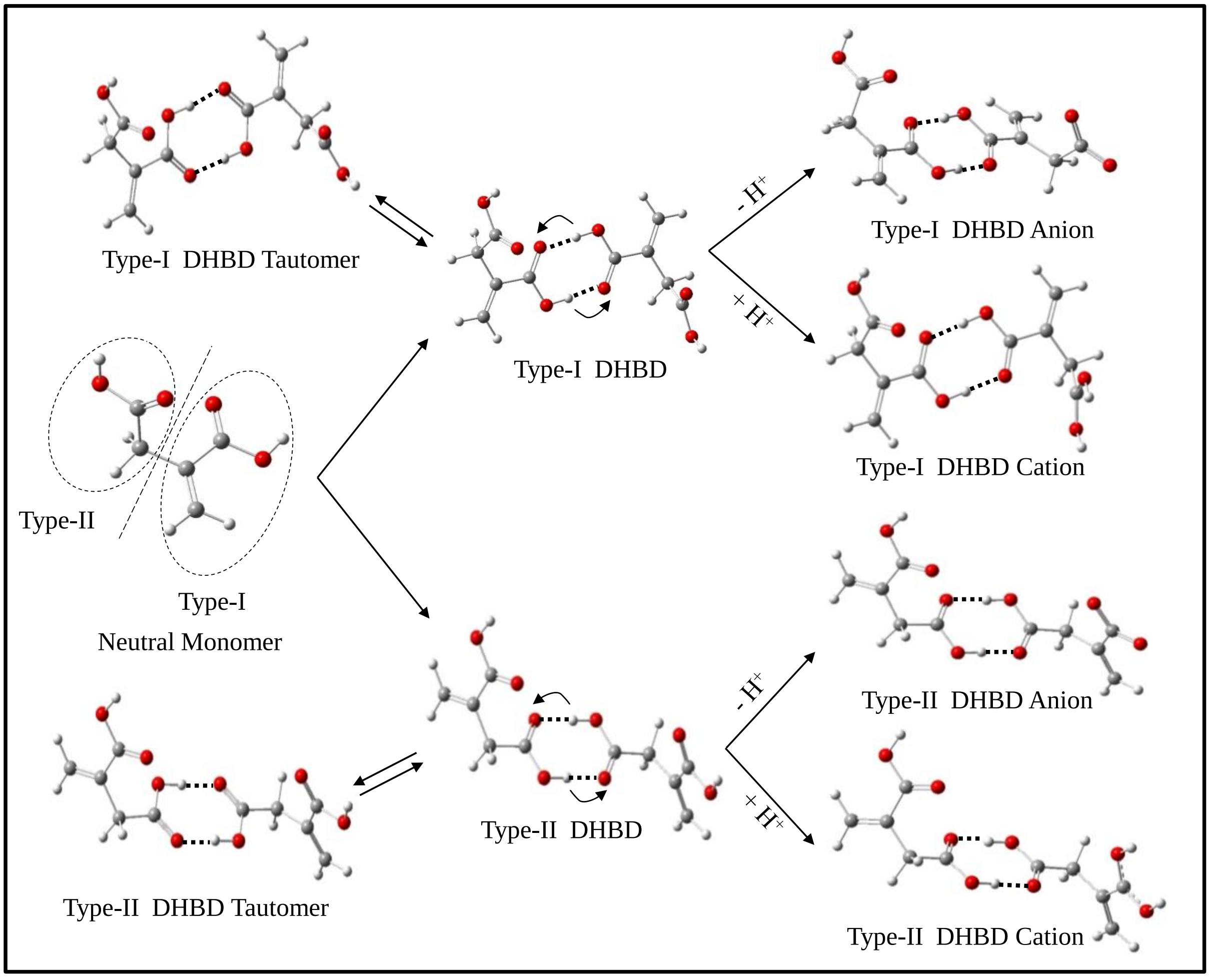

Figure S8: The PCM-B3LYP/6-31+G(d,p) level optimized geometries of doubly hydrogen-bonded dimers (DHBDs) and their ionic species considering the Type-I and Type-II carboxylic acid groups separately (see above).

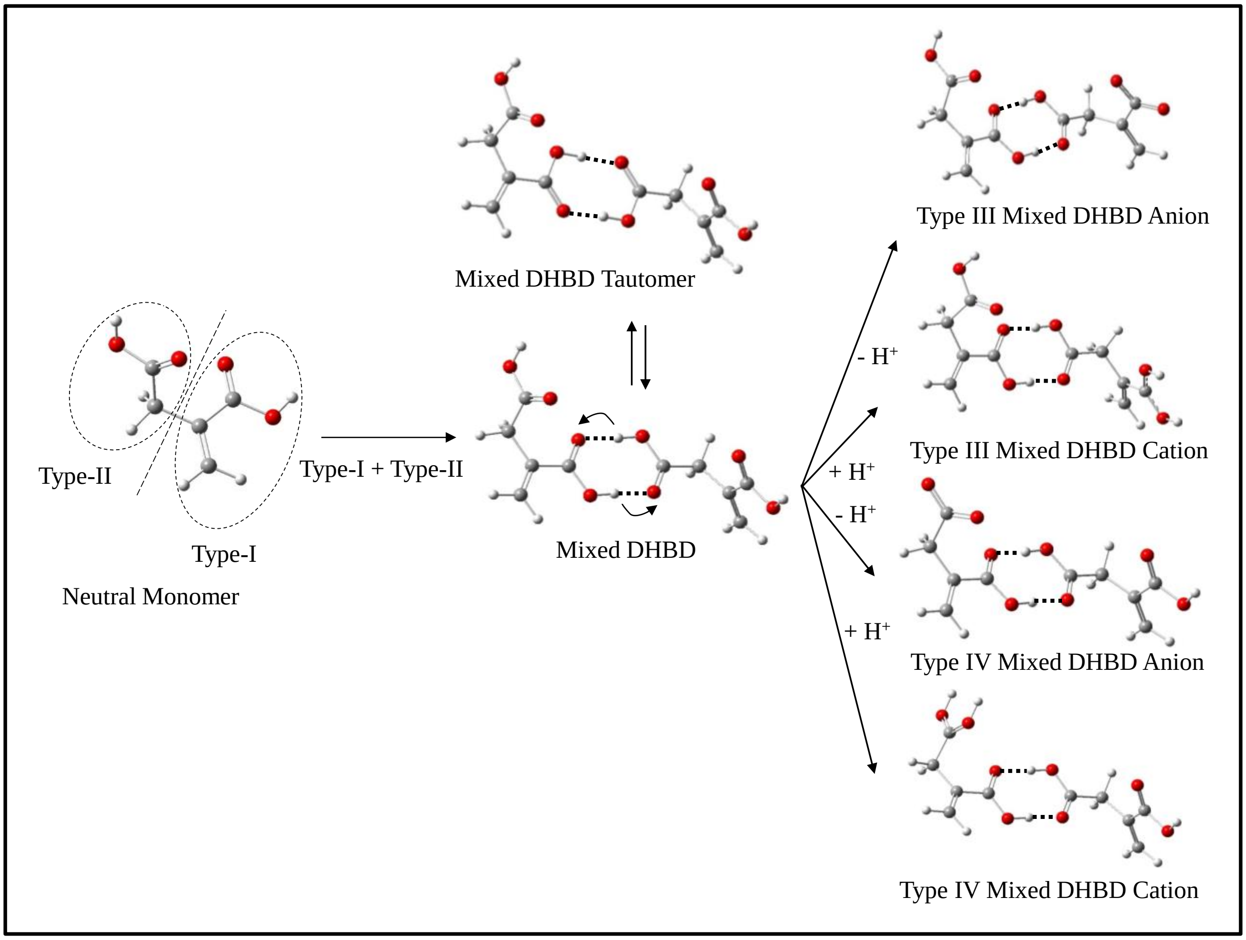

Figure S9: The PCM-B3LYP/6-31+G(d,p) level optimized geometries of mixed doubly hydrogen-bonded dimers (MDHBDs) and their ionic species considering the Type-I and Type-II carboxylic acid groups together (see above).

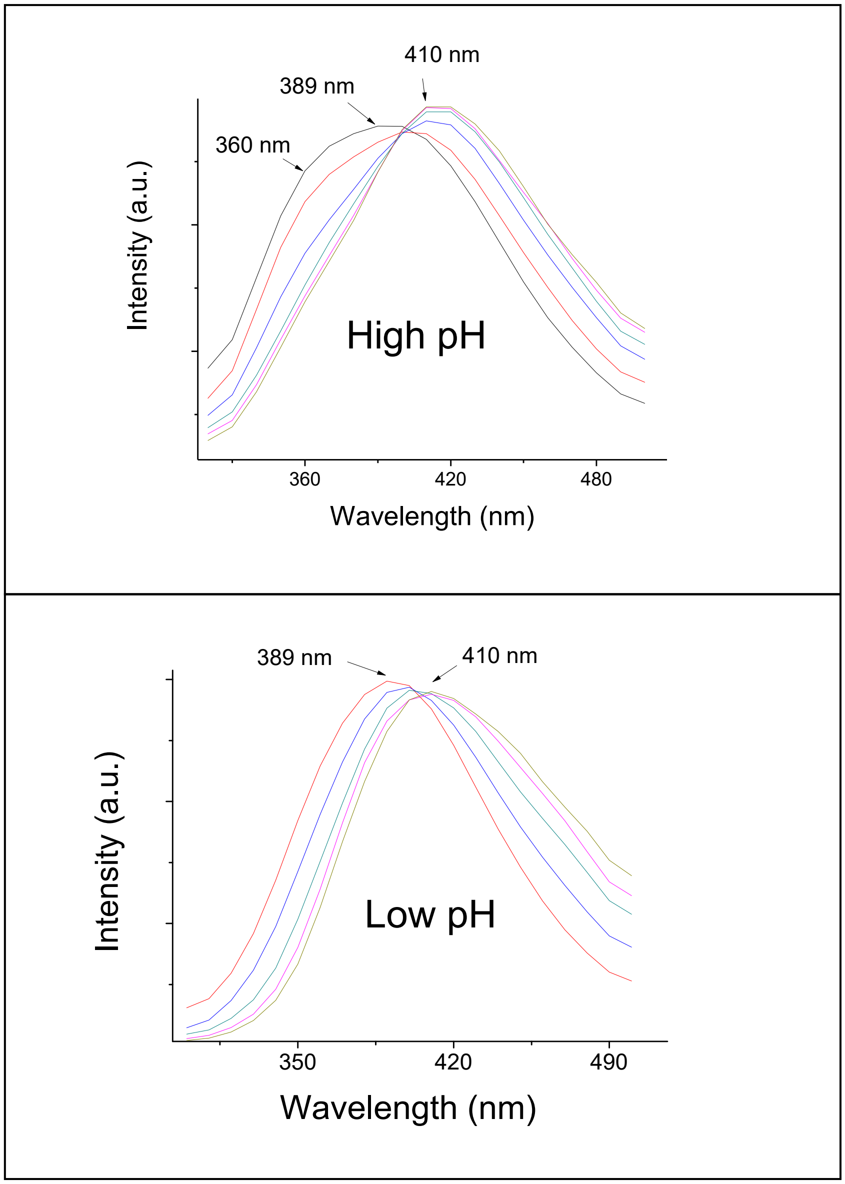

Figure S10. Time resolved area normalized emission spectra (TRANES) shows pH dependent spectral migration through isoemissive points. (pH 9-10 = High, pH 3-5 =Low)

References:

1. Frisch, M. J.et al. *Gaussian 09*, Revision B.01, Gaussian, Inc., Wallingford, CT, (2010).

2. D. Rappoport, F. Furche, Photoinduced Intramolecular Charge Transfer in 4-(Dimethyl)aminobenzonitrile- A Theoretical Perspective. *J. Am. Chem. Soc.* **126**, 1277- 1284 (2004).

3. C. Bernini, L. Zani, M. Calamante, G. Reginato, A. Mordini, M. Taddei, R. Basosi, A. Sinicropi, Excited State Geometries and Vertical Emission Energies of Solvated Dyes for DSSC: A PCM/TD-DFT Benchmark Study. *J. Chem. Theory Comput*. **10**, 3925- 3933 (2014).

4. R. Akbar, M. Baral, B. K. Kanungo, Experimental and DFT Assessment on the Development of Tris(methoxymethyl)-5-oxine. *J. Chem. Eng. Data.* **60**, 3236-3245 (2015).

5. B. J. Coe, A. Avramopoulos, M. G. Papadopoulos, K. Pierloot, S. Vancoillie, H. Reis, Theoretical Modelling of Photoswitching of Hyperpolarisabilities in Ruthenium Complexes. *Chem. Eur. J.* **19**, 15955-15963 (2013).

6. R. Improta, Towards effective and reliable fluorescence energies in solution by a new state specific polarizable continuum model time dependent density functional theory approach. *J. Chem. Phys.* **127**, 074504 (2007).

7. P. Zhou, J. Liu, S. Yang, J Chen, K. Han, G. He. The invalidity of the photo-induced electron transfer mechanism for fluorescein derivatives. *Phys. Chem. Chem. Phys.* **14,** 15191-15198 (2012).
